# Liver transplant donor-recipient matching with offline reinforcement learning

**DOI:** 10.1038/s41746-026-02529-1

**Published:** 2026-03-16

**Authors:** Andrew Melehy, Jeffrey Feng, Dominic Amara, Vatche G. Agopian, Alex A. T. Bui

**Affiliations:** University of California Los Angeles, Los Angeles, CA USA

**Keywords:** Computational biology and bioinformatics, Health care, Mathematics and computing, Medical research

## Abstract

Despite advances in liver transplantation (LT), deciding when to transplant a patient within the context of high waitlist mortality, organ scarcity, and risk of graft failure, remains an ongoing challenge. Existing approaches focus on the static prediction of successful LT donor-recipient pairs without weighing the competing interests such as the risk of graft failure against the risk of waitlist mortality and how these risks change over time. Instead, we used an offline reinforcement learning (RL) approach to represent the problem as the optimization of the series of decisions to wait, delist, or transplant a candidate at different timepoints. Using waitlist trajectories for LT candidates from the national Scientific Registry of Transplant Recipients (SRTR) database, we trained a model resulting in the avoidance of 73% of donor-recipient pairs that led to graft failure or death, preservation of 93% of successful transplants, and potentially suitable donors were found for 47% of those patients that died on the waitlist. Notably, the analysis of decisions and post-transplant survival revealed that our model learned features suggestive of successful donor-recipient pairs. Overall, we demonstrate how RL-based approaches better portray real-world LT donor-recipient matching decisions, illustrating their potential as useful clinical tools.

## Introduction

Liver transplantation (LT) is the gold standard treatment for end-stage liver disease^1^. Despite significant advances in the use of LT, waitlist mortality remains as high as 25%^2,3^. This high mortality is representative of several challenges, including donor scarcity and an aging recipient population with additional medical comorbidities that increase waitlist mortality risk. The model for end-stage liver disease (MELD) score, calculated from a candidate’s serum sodium, bilirubin, INR, and creatinine, is the standard for determining LT priority for patients with decompensated end-stage liver disease in the current United Network for Organ Sharing allocation policy^4–9^. Allocation policies (Share 35 in 2013, acuity circles in 2020) determine the offering order and are designed to increase the availability of organs to the most critically ill patients^10–14^.

When a donor organ is offered to a candidate, the patient’s transplant providers holistically review recipient and donor factors to determine the match suitability and mitigate the risk of graft failure. Despite an overall shortage of donor livers, many available donor livers are discarded or rejected by transplant teams due to a variety of reasons, including poor quality of the donor organ, concerns about donor-recipient size mismatch, or other logistical concerns^15^. Graft failure can be considered to include malfunction of the graft necessitating a new liver or death of the recipient. In either case, graft failure is a significant negative clinical event in LT due to the compromised health of that patient and the depletion of that organ from the donor pool. Graft failures occurring within the first year after transplant are thought to be due to donor-recipient match and operative characteristics, as many early graft failures are due to vascular compromise of the graft resulting from ischemia reperfusion injury, poor donor organ quality, recipient critical illness, among others^16,17^. Graft failures occurring later are more often due to organ rejection, complications resulting from immunosuppression, or recurrence of liver disease. Existing models can predict 3-month graft failure, like the survival outcomes following liver transplant (SOFT) and the balance of risk (BAR) score (area under the receiver operating curve; AUROC = 0.89)^18–20^. Likewise, several machine learning (ML) techniques predict early post-transplant graft failure with AUROCs from 0.90 to 0.96^21–26^.

However, existing predictive models only use data from a single time-point—often from donor and recipient features at time of transplant (e.g., SOFT and BAR)—to predict post-transplant mortality. These outcome-oriented models fail to evaluate dynamic changes (e.g., in MELD score and donor-recipient match suitability) over the course of the waitlist period and only consider transplant recipients—negating those patients who had waitlist mortality, an important aspect of real donor-recipient matching decisions.

Instead of conventional pattern recognition to predict outcomes from labeled data, offline reinforcement learning (RL) uses retrospective data to optimize the decisions throughout the LT donor-recipient matching process, given short- and long-term rewards^27–30^. Another advantage of offline RL is the ability to learn improved decisions when given retrospective examples of suboptimal trajectories, which is common in real-world data and in other domains (e.g., robotic control, self-driving, recommendation systems)^29,31–34^. Crucially, this approach better resembles the real decision-making of transplant providers compared to standard outcomes prediction, and considers the competing risks and benefits of multiple decisions at any given time for a donor-recipient pair, such as a transplant resulting in early post-LT graft failure or successful transplant vs. remaining on the waitlist resulting in recovery or mortality. While RL has been explored in many domains^35–48^, offline RL for medicine is an emerging field that has never been applied to optimize LT donor-recipient matching^49–59^. This application of offline RL has the potential to transcend pattern recognition towards a more accurate portrayal of real-world decision-making through the optimization of donor-recipient matching when accounting for multiple competing potential outcomes.

## Results

**Fig. 1:**
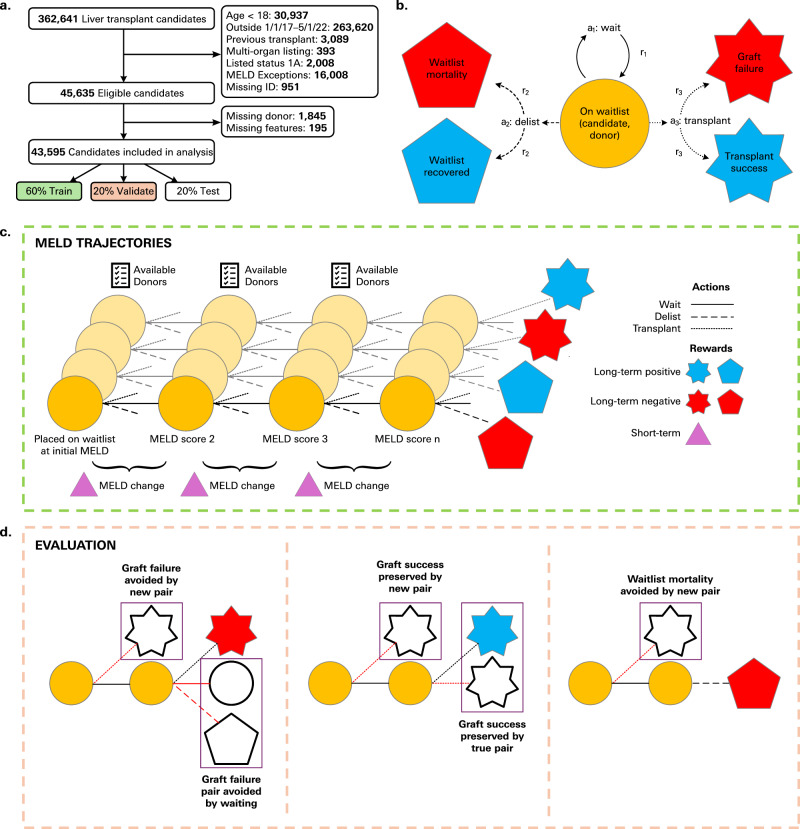
Overview of study and offline RL approach to LT donor-recipient matching. **a** Inclusion and exclusion criteria for arrival at the final cohort of patients used for analysis. **b** Markov decision process showing states derived from retrospective data (yellow: candidate and donor features with possible actions (transplant, delist, wait) that lead to discrete outcomes (blue: removal from the waitlist due to improved status resulting in a transplant no longer being required and transplant success; red: waitlist mortality and graft failure). **c** Training process diagram including visualization of MELD trajectories in sequence with possible donors at each point and potential actions available, which lead to one of four potential outcomes. The reward function is based on short-term (MELD change) and long-term (outcomes) rewards that are weighed together to optimize the decision-making process at each point in the trajectory. **d** Evaluation process diagram displaying the avoidance of graft failure by either transplanting that candidate to a different donor or point in the trajectory or by taking a new action (left panel), graft success preservation achieved by transplanting to true successful pair or the same candidate with a new pair (middle panel) and correct intervention where a waitlist mortality is avoided by selecting a potential donor for transplant (right panel).

This study used the Scientific Registry of Transplant Recipients (SRTR) database, with a full description of definitions, processing, and inclusion criteria in the Methods “Overview of the Scientific Registry of Transplant Recipients”. Of the 45,635 LT candidates who met the study inclusion criteria (Fig. 1a), 7616 (17%) died on the waitlist; 28,587 (63%) were transplanted, of whom 2119 (7%) had graft failure or died within 1-year post-transplant. Candidates’ demographic information satisfying the inclusion criteria is shown in Supplementary Table 1. After excluding cases for missingness (<5%), there were 43,595 candidates, 26,790 used donor organs, and 16,272 discarded organs available for analysis, resulting in 214,865 states after processing the episodes. Demographic comparisons between used and discarded donor livers are shown in Supplementary Table 2.

We processed each candidate’s MELD scores over time to generate episodes of an LT Markov Decision Process (MDP) (Fig. 1b), with complete details in Methods “Episode processing details”. A MELD score indicated a step in the MDP, with the candidate and potential donor’s features constituting the continuous state observations at that step, and the potential actions of waiting, delisting, or transplanting at each step (Fig. 1c). Rewards were associated with the state-action pairs and the outcomes of terminal transitions (details in Methods “Reward function design”). We used Conservative Q-learning (CQL), a popular standard in offline RL (Supplementary Fig. 3)^60,61^, with full details of the methods and experimental approach outlined in Methods “Conservative Q-learning” and Methods “Experimental pipeline”.

### Evaluation of reinforcement learning model performance

We aimed to understand how each model aligned with pragmatic clinical objectives of simultaneously preventing graft failures while maintaining transplants that led to (or could lead to) successful transplants and delisting candidates that recovered. Accordingly, we developed metrics contingent on observable actions and outcomes (Fig. 1d, Supplementary Table 3), with full definitions in Methods “Evaluation metrics”. To assess the ability to circumvent poor transplant outcomes, we measured “graft failure prevention rate” (GFPR) as the percent of real-world graft failure pairs avoided by the decision to not to transplant and “graft failure intervention rate” (GFIR) as the percent avoided by transplanting to a different pair. The retention of positive transplant outcomes was measured using “graft success preservation rate” (GSPR) as the percent of successful transplant candidates who were transplanted with their real-world or an earlier donor. The percent of recovered candidates who were chosen to be delisted was measured as the “correct removal rate” (CRR), and the percent of candidates who experienced waitlist mortality but were recommended earlier transplants was measured as the “correct intervention rate” (CIR). For each metric, we compared our learned model against baseline methods (Methods “Baseline comparisons to a MELD threshold” and “Baseline comparisons to static predictive models”), ranging from existing prioritization standards (i.e., MELD threshold model such as “MELD 15” that transplants any time a candidate’s MELD score exceeds 15) to static predictive models.

We selected a final offline RL model that was more conservative, indicating an ability to choose more optimal donor-recipient pairs, as opposed to more aggressive models attempting to maximize rewards by over-transplanting and ultimately selecting suboptimal pairs. In the holdout set, there were 5334 patients who received transplants in reality. The CQL model performed a mean total of 6927 (SD = 671.57) transplants in the holdout set compared to the indiscriminate MELD 15 model with 33,247 (SD = 0.00) transplants. Of the graft failure patients, the CQL model avoided the true donor-recipient pair in 73% (GFPR; SD = 2%) and selected a new donor-recipient pair for transplant in 55% (GFIR; SD = 3%). The model preserved 93% (GSPR; SD = 1%) of successful transplants by transplanting the true successful pair or by pairing the candidate to a new donor. The model correctly delisted 38% (CRR; SD = 3%) of patients who were delisted due to no longer requiring a transplant and transplanted 47% (CIR; SD = 3%) of patients who experienced waitlist dropout. Full metrics are reported in Table 1, with further subgroup analysis across demographic factors (candidate sex, location, and race) in Supplementary Table 4.

**Table 1:** Evaluation results

Model	Action match rate, % (SD)	Action: Wait, n (SD)	Action: Delist, n (SD)	Action: Transplant, n (SD)	GFPR, % (SD)	GFIR, % (SD)	GSPR, % (SD)	CRR, % (SD)	CIR, % (SD)
CQL	73.05 (0.97)	34,847.40 (733.34)	1,040.20 (116.31)	6927.40 (671.57)	73.18 (1.82)	54.74 (3.02)	93.09 (1.17)	37.55 (3.38)	46.83 (3.01)
Action PM: LGR	82.24 (0.00)	42,220.20 (0.84)	103.80 (0.45)	491.00 (0.71)	90.76 (0.00)	9.24 (0.00)	10.74 (0.02)	4.05 (0.00)	2.78 (0.00)
Outcome PM: LGR	34.49 (0.04)	10,582.20 (10.33)	3,323.40 (8.93)	28,909.40 (15.04)	14.69 (0.00)	97.63 (0.00)	99.87 (0.01)	73.88 (0.32)	91.62 (0.04)
Graft failure PM: LGR	51.61 (6.62)	24,359.20 (4121.84)	0.00 (0.00)	18,455.80 (4121.84)	45.02 (10.54)	76.35 (8.08)	87.81 (4.73)	0.00 (0.00)	78.94 (7.86)
Action PM: XGB	83.97 (0.11)	39,142.40 (36.20)	867.80 (34.84)	2804.80 (29.41)	64.74 (0.81)	40.24 (0.78)	79.68 (1.44)	36.20 (1.37)	10.81 (0.46)
Outcome PM: XGB	46.96 (0.30)	17,011.00 (170.59)	3,224.20 (19.51)	22,579.80 (187.39)	17.49 (1.36)	95.73 (0.87)	99.63 (0.06)	78.61 (0.38)	78.46 (0.84)
Graft failure PM: XGB	65.62 (8.51)	33,898.60 (4623.46)	0.00 (0.00)	8916.40 (4623.46)	72.18 (13.87)	55.02 (17.55)	78.35 (11.73)	0.00 (0.00)	58.50 (16.43)
MELD 15	38.86 (0.00)	9568.00 (0.00)	0.00 (0.00)	33,247.00 (0.00)	7.11 (0.00)	95.73 (0.00)	94.69 (0.00)	0.00 (0.00)	90.87 (0.00)
MELD 20	58.72 (0.00)	18,545.00 (0.00)	0.00 (0.00)	24,270.00 (0.00)	23.22 (0.00)	84.36 (0.00)	84.22 (0.00)	0.00 (0.00)	76.11 (0.00)
MELD 24	69.23 (0.00)	25,860.00 (0.00)	0.00 (0.00)	16,955.00 (0.00)	39.57 (0.00)	70.14 (0.00)	71.56 (0.00)	0.00 (0.00)	62.54 (0.00)
MELD 30	76.80 (0.00)	34,162.00 (0.00)	0.00 (0.00)	8653.00 (0.00)	61.61 (0.00)	46.68 (0.00)	48.90 (0.00)	0.00 (0.00)	41.03 (0.00)

Columns represent evaluation metrics: action match rate is the percentage of actions taken by the model that match what occurred in reality; action: wait is the number of times a decision was made to wait; action: delist is the number of times a decision was made to delist; action: transplant is the number of times a decision was made to transplant; GFPR is graft failure prevention rate or of the graft failure patents in what percentage of them did the model select a different action; GFIR is graft failure intervention rate or of the graft failure patients in what percentage of them did the model choose to transplant to a new donor; GSPR is the graft success preservation rate or in the successful transplant patients what percentage of them were transplanted by the model; CRR is the correct removal rate of the patients delisted due to no longer needing a transplant what percentage of them did the model choose to delist; CIR is correct intervention rate or of the waitlist mortality patients what percentage of them did the model choose to transplant. Rows represent model tested: CQL, a final conservative Q-learning based offline reinforcement learning model; PM, static predictive models trained on actions, outcomes, or transplant outcomes using logistic regression (LGR) or eXtreme gradient boosting (XGB); MELD #, MELD threshold models using thresholds 15, 20, 24, and 30.

### Features indicative of successful donor-recipient pairs

We compared the feature distributions of the counterfactual donor-recipient matches with respect to successful donor-recipient pairs to interpret their clinical feasibility^62^. These comparisons were conducted in three subgroups of candidates who (in reality): (1) experienced graft failure; (2) experienced waitlist mortality; or (3) were transplanted successfully, but the model selected a new donor-recipient pair. We compared the feature distributions of discarded organs that the model chose to transplant vs. not transplant to observe if our model was tending towards certain donor dispositions in counterfactual transplants, with statistical tests described in Methods “Feature comparisons”.

**Fig. 2:**
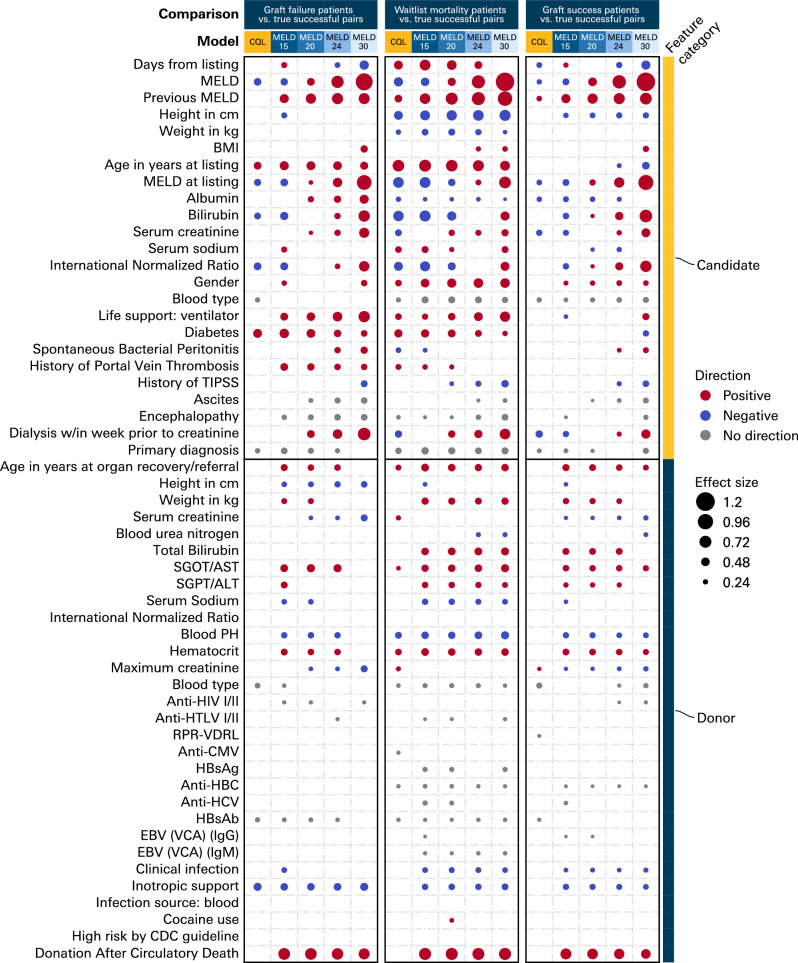
Feature maps comparing variables of new donor-recipient pairs selected by various models (columns) in: true graft failure candidates to true successful donor-recipient pairs (left); true waitlist mortality candidates to true successful donor-recipient pairs (center); and true graft success candidates (new selected donor/ point in MELD trajectory) to true successful donor-recipient pairs (right). If a box is white there was no significant difference in that feature (reference: true successful donor-recipient pairs). Red indicates a positive feature difference, blue a negative, and gray a non-directional effect (from comparing multi-category variables). Circle size indicates the magnitude of the effect size.

The counterfactual pairs selected by the CQL model did not significantly differ from true successful donor-recipient pairs with respect to number of donations after circulatory death (DCD) donors, donor pH, sodium, and transaminase levels, candidate encephalopathy, need for dialysis, and need for mechanical ventilation (Fig. 2). Pairs selected by the model tended to have lower candidate MELD scores. In contrast, the MELD threshold models chose pairs with higher MELD scores, more candidates requiring mechanical ventilation and dialysis, candidates more likely to have portal vein thrombosis and bacterial peritonitis, and poor-quality donors in general (more DCD, higher transaminase, lower sodium, lower pH, higher bilirubin). Remarkably, the final CQL model utilized 461 (23%) discarded organs that shared characteristics of relatively higher donor quality compared to other discards (fewer DCD, less likely to have an infection, lower transaminase levels, higher sodium, and pH; Supplementary Fig. 1). We also illustrate examples of simulated case-level trajectories that indicate transplant decisions being made when there is low candidate MELD, higher donor PH, lower donor creatinine, lower donor age, and a closer match between donor and candidate weight and height (Supplementary Fig. 4).

### Differentiation of survival in real-word donor-recipient pairs

**Fig. 3:**
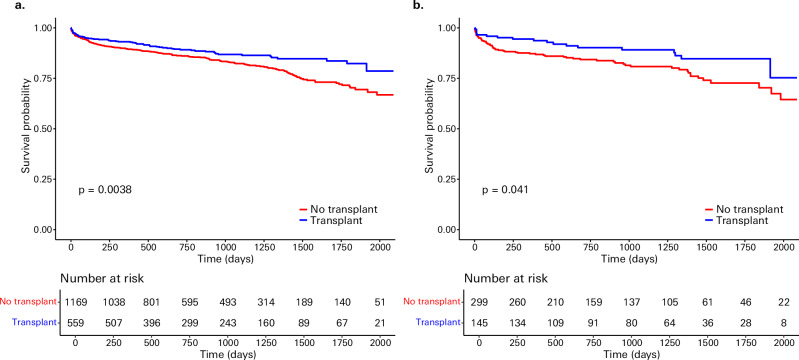
Post-transplant survival comparisons between those chosen for transplant by the model and those not chosen. Specifically, these Kaplan–Meier curves compare post-transplant survival in: **a** true donor-recipient pairs within those designated high-moderate, high, and futile by the Survival Outcomes Following Liver Transplantation (SOFT) score; and **b** true donor-recipient pairs within those designated high-risk by the Balance of Risk (BAR) score (BAR ≥ 18). Those selected for transplant by the model are shown in blue and those not transplanted shown in red. Survival differences were assessed using log-rank tests with p-values less than 0.05 considered statistically significant.

We compared how our model discriminated post-transplant survival for those who received a transplant in real-life vs. SOFT (high-moderate, high, and futile vs. low and low-moderate) and BAR (BAR score ≥18 vs. <18) scores. For patients indexed within the high-risk category of the SOFT/BAR scores, we determined if the patients that our model chose to transplant vs. not transplant further differed in survival. In real-world donor-recipient pairs, there was a significant post-transplant survival advantage in patients that the model chose to transplant vs. those it chose not to (*p* < 0.001) (Supplementary Fig. 2a). In comparison, high-risk by SOFT was associated with decreased post-transplant survival in our cohort (*p* = 0.01), while the BAR score was not (*p* = 0.11) (Supplementary Fig. 2b, c). Even within high-risk patients by these scores, our model transplanted pairs with improved post-transplant survival (SOFT, *p* = 0.004; BAR, *p* = 0.04) (Fig. 3a, b). Notably, the median SOFT and BAR scores between patients transplanted vs. not transplanted by the model in the high-risk subset of each score did not significantly differ (SOFT *p* = 0.20; BAR *p* = 0.80). Note that this analysis was limited to the true donor-recipient pairs that occurred; in a full simulation, the model may transplant these candidates earlier in their trajectories and/or to different donors rather than not transplant them.

### Application in a constrained setting

We also simulated the models in a setting with more realistic resource constraints (Methods “Evaluation in a constrained setting”). In this constrained setting, where each candidate and donor would be removed from analysis following transplant, death, or removal, the model transplanted 86% of graft failure patients to a different donor. Furthermore, 85% of patients who faced waitlist mortality were transplanted to possible donors, and 90% of candidates who had successful donor-recipient pairs were transplanted (Table 2). The MELD 15 threshold model transplanted 95% of graft failure patients to new donors, 91% of waitlist mortality patients, and 95% of successful transplant patients. But it achieved these results by naively transplanting nearly every patient (i.e., when MELD ≥ 15) and chose suboptimal donor-recipient combinations (Fig. 2).

**Table 2:** Constrained evaluation results

	Action			Graft failures				Successful transplant		Waitlist recovered		Waitlist mortality	
Model	Wait, n (SD)	Delist, n (SD)	Tx, n (SD)	Tx, % (SD)	Delist, % (SD)	Potential intervention, % (SD)	Incorrect intervention, % (SD)	Tx, % (SD)	Delist, % (SD)	Tx, % (SD)	Delist, % (SD)	Tx, % (SD)	Delist, % (SD)
CQL	784.20 (116.89)	553.00 (26.55)	7381.80 (122.99)	89.67 (1.36)	2.27 (0.70)	86.45 (1.70)	3.22 (0.74)	89.82 (1.10)	2.22 (0.17)	67.12 (2.84)	22.98 (1.41)	84.59 (1.90)	5.92 (0.61)
Action PM: LGR	8032.20 (0.84)	91.00 (0.00)	595.80 (0.84)	10.43 (0.00)	0.00 (0.00)	3.32 (0.00)	7.11 (0.00)	9.23 (0.02)	0.14 (0.00)	0.47 (0.00)	6.31 (0.00)	5.59 (0.04)	0.46 (0.00)
Outcome PM: LGR	48.00 (0.00)	244.80 (4.76)	8426.20 (4.76)	98.91 (0.13)	0.85 (0.13)	98.44 (0.13)	0.47 (0.00)	99.12 (0.03)	0.72 (0.03)	88.96 (0.15)	10.56 (0.15)	95.63 (0.07)	2.85 (0.07)
Graft failure PM: LGR	1455.80 (630.84)	0.00 (0.00)	7263.20 (630.84)	88.91 (4.64)	0.00 (0.00)	86.97 (4.94)	1.94 (0.31)	82.00 (7.93)	0.00 (0.00)	80.32 (9.07)	0.00 (0.00)	91.33 (4.12)	0.00 (0.00)
Action PM: XGB	2435.60 (154.56)	668.60 (24.28)	5614.80 (177.72)	75.26 (2.41)	2.84 (0.75)	65.26 (2.26)	10.00 (0.81)	75.12 (1.42)	1.60 (0.15)	31.26 (3.64)	35.54 (1.21)	60.97 (2.35)	8.33 (0.28)
Outcome PM: XGB	70.00 (2.45)	466.80 (3.35)	8182.20 (3.03)	97.91 (0.26)	1.99 (0.13)	97.35 (0.39)	0.57 (0.13)	98.05 (0.05)	1.71 (0.02)	79.08 (0.38)	19.57 (0.36)	93.95 (0.33)	4.67 (0.23)
Graft failure PM: XGB	2074.80 (1062.03)	0.00 (0.00)	6644.20 (1062.03)	81.66 (9.17)	0.00 (0.00)	80.24 (9.26)	1.42 (0.29)	73.80 (12.46)	0.00 (0.00)	75.54 (15.05)	0.00 (0.00)	84.65 (9.17)	0.00 (0.00)
MELD 15	1048.00 (0.00)	0.00 (0.00)	7671.00 (0.00)	95.97 (0.00)	0.00 (0.00)	95.26 (0.00)	0.71 (0.00)	94.61 (0.00)	0.00 (0.00)	61.39 (0.00)	0.00 (0.00)	90.73 (0.00)	0.00 (0.00)
MELD 20	2334.00 (0.00)	0.00 (0.00)	6385.00 (0.00)	84.60 (0.00)	0.00 (0.00)	83.41 (0.00)	1.18 (0.00)	84.18 (0.00)	0.00 (0.00)	32.02 (0.00)	0.00 (0.00)	76.04 (0.00)	0.00 (0.00)
MELD 24	3594.00 (0.00)	0.00 (0.00)	5125.00 (0.00)	70.38 (0.00)	0.00 (0.00)	68.96 (0.00)	1.42 (0.00)	71.50 (0.00)	0.00 (0.00)	13.28 (0.00)	0.00 (0.00)	62.41 (0.00)	0.00 (0.00)
MELD 30	5394.00 (0.00)	0.00 (0.00)	3325.00 (0.00)	46.68 (0.00)	0.00 (0.00)	44.55 (0.00)	2.13 (0.00)	48.88 (0.00)	0.00 (0.00)	2.64 (0.00)	0.00 (0.00)	40.97 (0.00)	0.00 (0.00)

Columns represent evaluation metrics with the total number of actions (standard deviation) taken by each model (left panel), specific actions in graft failure patients, including the percentage chosen for transplant (Tx) and delisting. Potential intervention refers to the percentage of graft failure patients transplanted to a new donor, and incorrect intervention the percentage of patients transplanted to the same donor that led to the graft failure (middle panel). The percentage of patients transplanted and delisted by each model in successfully transplanted patients, those who were delisted and recovered, and those who experienced waitlist mortality is shown in the three right panels. Rows represent model tested: CQL, a final conservative Q-learning based offline reinforcement learning model; PM, static predictive models trained on actions, outcomes, or transplant outcomes using logistic regression (LR) or eXtreme gradient boosting (XGB); MELD #, meld threshold models using thresholds 15, 20, 24, and 30.

## Discussion

In this work, we approached the optimization of decisions in LT donor-recipient matching using offline RL. Our approach utilized the interplay of the many factors that affect the risk of graft failure, including donor-recipient characteristics, candidate trajectories, and transplant timing. In contrast, the static predictive models could prevent poor outcomes (graft failure in the action prediction model; waitlist mortality in the outcome prediction model), but only at the expense of another outcome (increased waitlist mortality in the action prediction model; graft failure in the outcome prediction model). The graft failure prediction model balanced the identification of successful transplants and the avoidance of graft failures, but performance was still worse than the offline RL model. Overall, static predictive models are limited to the correlation of features with an outcome, as opposed to accounting for multiple competing considerations involved in longitudinal processes of real-world decision-making. Similarly, in the MELD threshold models, we observed that with increasing threshold, graft failure was reduced by transplanting less, and with a decreasing threshold, waitlist mortality was reduced by transplanting more; the outcomes cannot be optimized simultaneously, only based on candidate acuity.

As graft failure could be entirely avoided by choosing to never transplant, the prevention of graft failure must be contextualized alongside the preservation of successfully transplanted candidates. Thus, our model’s ability to preserve 93% of successfully transplanted candidates while avoiding graft failure in 73% of patients is significant. Additionally, waitlist dropout challenges LT, with many candidates becoming ineligible due to severe clinical decompensation, and others being severely ill at the time of listing, such that transplant would not result in an acceptable outcome regardless of timing or donor. Of patients who experienced waitlist death or delisting, our model selected 47% to receive a transplant, suggesting that it identified donors and times in candidate trajectories that would maximize the likelihood of successful transplant. Markedly, existing models to predict graft failure (SOFT and BAR) are limited in their examination of only true donor-recipient pairs, precluding their application to real-world LT and considerations of waitlist mortality. While we cannot directly compare to the SOFT and BAR scores, our model differentiates survival in recipients within the high-risk classification of SOFT and BAR. Remarkably, these survival differences were not associated with differences in the values of either score, indicating that our model did not simply identify patients with lower SOFT and BAR scores for transplant, but recognized complexities indicative of improved survival that the SOFT and BAR scores were unable to discern.

A goal of offline RL is to learn counterfactual decisions (e.g., donor-recipient pairs that did not occur). The MELD threshold models serve as an important control in this light, given that they know nothing about donor-recipient pair quality and consistently transplant when the MELD score of a candidate exceeds a threshold. This analysis shows that the pairs selected by the model were of better disposition compared to the pairs selected by the MELD threshold models, with fewer feature differences with successful donor-recipient pairs. Our model also identified discarded donors that were more likely to be suitable for transplant. Notably, the reward function design assumed that these organs were non-randomly discarded and were, on average, worse than those used in transplants. The model thus determined that the likelihood of a successful transplant for a given candidate outweighed the risk of using a discarded organ, demonstrating a learned complexity regarding the optimization of several considerations when selecting a donor-recipient pair. Moreover, this finding highlights the potential for such a model to theoretically reduce the number of discarded livers by assisting in the selection of likely successful pairs.

In our primary analysis (Table 1), there was potential for the same donor to be offered to multiple candidates. The constrained analysis removes donors when they are transplanted and candidates after they are delisted or transplanted. Although this analysis lacks true real-world constraints, it offers important insight by more closely approximating reality. The constrained model chose to transplant 90% of those experiencing graft failure; in reality, 90% of those who had true successful transplants, and 85% of those who experienced waitlist mortality. These results suggest that the model continues to optimize donor-recipient matching to reduce both graft failure and waitlist mortality and that, with an ample supply of donors, successfully matching nearly every patient is theoretically possible. Still, an important caveat is that even in this “constrained” analysis, the donor pool was far less restricted than in reality.

The results are promising in terms of reducing waitlist mortality and early post-transplant mortality and are robust with minimal variability across cross-validation folds. Additionally, the correlation with real post-transplant survival differences between patients it selects for transplant having better post-transplant survival than those it does not improves our confidence in this approach. Our work demonstrates promising preliminary insight in the potential for an offline RL approach to address the LT donor-recipient matching problem, but it is not intended for operational use in its current form. There are several limitations that we summarize into four main themes for future work that are important requisites to improving real-world utility:

1. Incorporating additional real-world constraints such as those related to SRTR regions. To maximize the learning of meaningful donor-recipient matches, we did not incorporate the geographical restrictions considered in the true allocation policy^10–14^. However, differences in donor availability, demographics, and transplant practices across SRTR regions suggest a potential trade-off between optimizing a global model vs. tailoring to specific locations. Additionally, techniques like normothermic machine perfusion will continue to change the landscape of organ availability and allocation policy, particularly as it pertains to DCD donor allografts^3^. Due to the study period ending in May 2022, machine perfusion did not have a significant impact on the present study (1.5% of transplanted donors and <1% of discarded donors), but could be explored in future work once the data reflects wide adoption of this practice. There is also an opportunity to explore inter-site variability, optimize allocation in the context of multiple health systems in a unified network, and validate the offline RL approach in countries with different allocation policies. While we demonstrated that results remained largely consistent with slight variation across regions and demographics, future work will incorporate these factors and constraints, especially regional constraints to reflect real-world allocation and geographical limitations, as we move towards creating a comprehensive and pragmatic clinical tool.
2. Collecting high-quality prospective data that better reflects real-world decisions over time. Given the limitations of the SRTR dataset and assumptions required in this study, it will be necessary to collaborate with clinicians and collect prospective data that ascertains the real-life patterns of sequential decision-making in LT donor-recipient matching. A core assumption in this work was the existence of unobserved decisions – that offers were made but declined throughout the duration of a candidate’s time on the waitlist. While reasonable to some degree, the extent to which this assumption holds is impossible to verify in the SRTR dataset. The added perspective of how clinicians balance short- and long-term risks when making decisions will allow us to refine training and improve evaluation to ensure efficacy and alignment with clinical goals. Furthermore, granular documentation of reasons for outcomes such as waitlist death would also provide better real-world context for clinicians’ decisions.
3. Exploring alternative algorithms and their interpretability. To illustrate the value of the RL approach, we maintained simplicity in elements such as the Q-function network architecture, reward function, and MDP. There remains a vast optimization space to be fully explored and the potential for additional hyperparameter tuning in future work. There is also strong clinical motivation for accompanying explanations to assess the trustworthiness of model-based decisions. Along with these modeling advancements, future work will explore the ability to improve case-level interpretability through approaches such as feature-based explanation methods and Shapley values for RL^63^.
4. Aligning with real-world settings to improve decision-making. The model is currently framed as a decision support tool where, when a specific candidate is offered a potential donor liver, a recommendation to transplant, wait, or delist is given. This assessment is based on the candidate trajectory and candidate and donor features, from which the model has learned to minimize waitlist mortality and early post-transplant mortality simultaneously. However, we assumed that decisions were made independently between single candidate-donor pairs. Modeling how one organ can potentially be offered to multiple candidates (e.g., in a multi-agent system) will better represent the donor scarcity problem. Such an approach can also incorporate inter-candidate dependency, as clinicians do not reach conclusions in isolation, and decisions made for one candidate inevitably impact the potential outcomes for other patients. In reality, donor scarcity and disparities in access also exacerbate concerns related to fairness and equity of allocation. In future work, we will extend our stratified analysis to explore potential disparities and solutions through calibration and fair RL methods^64^.

Overall, we applied offline RL to represent the clinical decision-making process involved in LT donor-recipient matching, improving upon existing methods for organ allocation by reducing the rate of graft failure while preserving the rate of successful transplants. While this work provides preliminary insight, re-examining how data-driven methods are applied to donor-recipient matching through the lens of RL shows potential in advancing the design and implementation of clinical decision-making tools. Given the promising directions of current findings, we believe this motivates the exploration of next steps that bring the developed models closer to real-world use.

## Methods

### Overview of the scientific registry of transplant recipients

This study used the SRTR database, which includes data on all donor, waitlisted candidates, and transplant recipients in the United States (US) (full description in Data Availability). We identified adult (age >18), first-time, non-status 1 A, liver-only waitlist candidates from 1/1/2017-5/1/2022 (Fig. 1a). A subset of candidates who received MELD exception points to increase their prioritization (most commonly for hepatocellular carcinoma) were excluded from analysis. The defined outcomes were waitlist death or delisting due to medical deterioration or being “too sick for transplant,” delisting due to medical condition improvement with no further need for LT, successful transplant, or graft failure (need for re-transplant), or mortality within 1 year of LT. Graft failure or all-cause mortality within 1 year is a well-established post-LT endpoint^20,21,65–67^. Patients who remained on the waitlist during the study period were included in the analysis with no defined outcome. Clinical, laboratory, and demographic features were captured at the time of listing, and all reported MELD scores were collected from listing to waitlist outcome. Additionally, discarded donors with a discard date within the timeframe above were included in this study. Candidates were excluded if there was a documented date of transplant with no corresponding donor information, or they had missing features. This study involved secondary analysis of de-identified registry data and was granted exemption from Institutional Review Board approval at the University of California, Los Angeles. All research was done in accordance with the Declaration of Helsinki.

### Episode processing details

In this study, each of the 43,595 candidates exhibited a unique trajectory of MELD scores over time, resulting in the generation of 43,595 processed episodes with a mean of 4.93 (SD = 3.36) MELD scores per episode. Thus, the total number of unique candidate-MELD instances was 214,865, with each MELD score indicating a step in an LT MDP (Fig. 1b). The possible actions at each step were to: (1) *wait* (i.e., the candidate remaining on the waitlist); (2) *delist*; or (3) *transplant*. Delist/transplant were terminal actions that marked the end of an episode. In the SRTR dataset, only terminal actions were observed at the final MELD scores for each candidate, and donor-recipient matches were indicated for transplants that occurred. Therefore, we assumed that obtaining an earlier MELD score was associated with the decision to keep a candidate on the waitlist, and we inferred state transitions based on potential available donors at that time. For every non-transplant step in the MDP, we identified the set of actual donors and discarded organs with a recovery date closest to the time of each MELD score; if there were multiple potential donors, one was randomly sampled to represent the state antecedent to the action at that step. The set of potential donors was restricted to those that matched the blood type of the candidate. Multiple patients with the same blood type and MELDs recorded on the same day could be offered the same donor. Altogether, the observations at each state throughout a candidate’s episode were represented by their static listing features, changing MELD over time, and a new potential donor organ at each step.

One potential donor randomly sampled at each time of MELD for each candidate resulted in 214,865 as the exact number of states and transitions to be used in the offline RL analysis. However, this subset was only one instantiation of the total number of possibilities. At each time of MELD, there was a mean of 8.09 (SD = 4.60) potential donors, including discarded organs. Therefore, the total number of possible states, including all combinations of candidates, times of MELD, and potential donors, was on the order of 1.74 M. When considering all possible states, the number of transitions was on the order of 13.3 M. There were limitations in how our sampling applied a simplifying assumption on the dataset that deviated from the real-life decision-making process (i.e., we sampled one possible decision with one potential match at a time), and pose future work in the direction of representations that better align with reality.

### Reward function design

While the design of reward functions is understood to often require a trial-and-error approach^68–70^, we followed best practices and exemplars from existing RL examples in healthcare^37,40,43,48,51^. The rewards were designed to reflect a clinician’s perspective of a patient’s condition while on the waitlist, as well as their final outcome. As illustrated in Fig. 1b, there were three components to the reward function, corresponding to the possible actions at each step in an episode. The reward for deciding to wait and remain on the waitlist is described in Eq. 1, where *i* indicates the step in the episode. A decrease/increase in MELD associated with improving/worsening patient condition was used as a positive/negative reward. The magnitude of MELD change was scaled by a factor of how close the MELD was to 24 (the median MELD score in the study population). The ${1}_{A}(x)$ denotes the indicator function that provided additional reward of ${\beta }_{1}$ and ${\beta }_{2}$, respectively, when waiting on potential donors that led to eventual graft failures or were eventually discarded. These rewards employed the assumption that, on average, such organs can be expected to be lower quality than those that were used, thus guiding the model to learn more optimal donor-recipient matches. As there are many reasons for discarding organs, we asserted that ${\beta }_{2} < {\beta }_{1}$ to reflect that they should be generally avoided in transplants, but less so than organs that definitively resulted in graft failure. In general, graft failure is the worst outcome due to the effective loss of at least two lives, as the decision to transplant is nearly never independent of the decision not to transplant another patient.

1 $${r}_{1,i}=\left({\mathrm{MELD}}_{i}-{\mathrm{MELD}}_{i+1}\right)\times \frac{min({\mathrm{MELD}}_{i+1},24)}{24}+{\beta }_{1}{1}_{\mathrm{GFdonor}}\left(\mathrm{donor}\right)+\,{\beta }_{2}{1}_{\mathrm{Discarded}\mathrm{donor}}\left(\mathrm{donor}\right)$$

The reward for delisting a patient from the waitlist was dependent on the outcome for the patient (Eq. 2). If patients were delisted due to medical deterioration or being too sick to transplant, the action was penalized with ${\beta }_{3}$ to steer the model to alternative outcomes. Delisting for medical condition improvement was rewarded with ${\beta }_{4}$ as a positive outcome.

2 $${r}_{2}=\left\{\begin{array}{l}{\beta }_{3}\,\mathrm{if}\,\mathrm{died}\\ {\beta }_{4}\,\mathrm{if}\,\mathrm{recovered}\end{array}\right.$$

The reward for transplant was based on the outcome of the operation (Eq. 3). Transplants that resulted in a 1-year graft failure were penalized with ${\beta }_{5}$ and transplants that led to successful transplants were rewarded with ${\beta }_{6}$.

3 $${r}_{3}=\left\{\begin{array}{l}{\beta }_{5}\,\mathrm{if}\,\mathrm{graft}\,\mathrm{failure}\\ {\beta }_{6}\,\mathrm{if}\,\mathrm{successful}\,\mathrm{transplant}\end{array}\right.$$

The final reward function was the sum of the constituents in Eqs. 1–3. Overall, positive rewards from terminal actions were associated with successful transplants and delisting due to candidate recovery; negative rewards were attributed to graft failures and death on the waitlist. Remaining on the waitlist resulted in a reward dependent on the subsequent change in MELD, as well as a general notion of donor quality; remaining on the waitlist when potentially matched with a discarded organ was associated with a positive reward; and a greater reward when with a donor that resulted in graft failure. We examined the sensitivity of reward function parametrizations by performing a grid search over: ${\beta }_{1}=\left\{1.0,\,0.5\right\}$, ${\beta }_{2}=\left\{\mathrm{0,0.1},\,0.25,\,0.5\right\}$, ${\beta }_{3}=\left\{-1.0\right\}$, ${\beta }_{4}=\left\{1.0\right\}$, ${\beta }_{5}=\left\{-2.0,\,-4.0,\,-8.0\right\}$, ${\beta }_{6}=\left\{1.0,\,2.0\right\}$. Our main interests (and hence focus on ${\beta }_{2}$ and ${\beta }_{5}$) were exploring the extent of contribution of including the discarded organs in the analysis (which has not been explored in this domain), and the level of penalization to apply to graft failures. The optimal parameterization was: ${\beta }_{1}=1.0$, ${\beta }_{2}=0.25$, ${\beta }_{3}=-1.0$, ${\beta }_{4}=1.0$, ${\beta }_{5}=-4.0$, ${\beta }_{6}=1.0$. Details of reward design and components are summarized in Supplementary Table 5.

### Conservative Q-learning

Offline RL methods share the problem of extrapolation error due to distribution shift between the learned model and the underlying behavior policy that collected the training data^28–30,71–73^. Despite new approaches in both of the two key branches of techniques for offline RL (model-free vs. model-based), CQL remains a popular standard due to its strong performance on conventional benchmarks and practical implementation. The CQL algorithm combines the conventional Q-learning objective with an additional term for regularization controlled by a hyperparameter, *α* (Eq. 4). Intuitively, the regularization term minimizes large Q-values and thereby avoids the over-estimation of out-of-distribution actions. CQL extends conventional RL with deep Q-networks (DQN), which uses neural networks to model the action-value function, allowing for continuous states. Not only do we utilize double deep Q-networks (DDQN) to mitigate overestimation errors, but we also use a modified encoder of the state space that applies late fusion to candidate and donor embeddings generated by upstream neural networks. We used a simple architecture consisting of two hidden layers with 256 units and rectified linear unit (ReLU) activation functions. To consolidate the different candidate and donor features, we incorporated separate upstream encoders that embed the different feature vectors into a denser representation and applied late fusion to form the final embedding for Q-learning (Supplementary Fig. 3).

4 $$L(\theta )={\rm{\alpha }}{E}_{s{\mathscr{\sim }}{\mathscr{D}}}\left[\log {\sum }_{a}\exp \left(Q\left(s,a\right)\right)-{E}_{a{\mathscr{\sim }}{\mathscr{D}}}\left[Q\left(s,a\right)\right]\right]+{L}_{\text{Double Deep Q}-\mathrm{learning}}(\theta )$$

We trained all models with 1.25 M iterations at a batch size of 256. Given that *α* represents how conservative the learned model should be, we tuned this parameter across $\alpha =\left\{0,\,0.001,\,0.01,\,0.1,\,0.5,\,1.0\right\}$, resulting in an optimal value of $\alpha =0.1$.

### Experimental pipeline

To derive the final model, we applied a systematic approach of learning and validation. First, we split the dataset into 80/20 training and holdout test cohorts. On the training cohort, we performed 5-fold cross-validation to identify the optimal hyperparameters as well as assess sensitivity across different randomizations of the training data. The models trained on each fold were evaluated on the holdout set to obtain standard deviations of the performance metrics. For the downstream interpretation of the models, we retrained a single model with the optimal configuration identified during cross-validation. This final model was used to perform any analysis on the feature distributions and mortality within the holdout set. Finally, we used the optimal configuration to assess sensitivity to reward function parameterizations.

### Evaluation metrics

A challenge of offline RL is evaluating the learned models, as a given result can be counterfactual and non-existent in the retrospectively collected data. To conduct offline RL evaluation, we relied on metrics that offered clinical interpretations of the models. These domain-centric metrics better exhibit the value of the models with respect to clinical objectives and expectations.

As the only observable negative outcome of a transplant was graft failures, we explicitly evaluated whether the model decided against transplanting the true pairs to provide insight into the learning of “bad” matches. Specifically, given the true donor-recipient pairs from the candidates of the holdout set that experienced graft failure, we defined “graft failure prevention rate” (GFPR) as the percent that the model decided not to transplant. Beyond explicitly evaluating the true pair, we also assessed the ability to “avoid” a graft failure by transplanting a new pair resulting in potential improvements to clinical outcomes. We defined “graft failure intervention rate” (GFIR) as those who were matched with earlier donors, summarized as the percent of candidates who experienced graft failure that were matched with an alternative donor. Conversely, observable positive outcomes were successful transplants, and we evaluated if candidates from these pairs were transplanted with their true donor or an earlier donor, assuming that they were amenable to other potential donors. Given real-life or earlier pairs from the candidates of the holdout set that experienced a successful transplant, we defined “graft success preservation rate” (GSPR) as the percent that the model decided to transplant. Both GFPR and GSPR are forms of sensitivity.

For candidates who experienced waitlist mortality, the percentage that were recommended alternative interventions as earlier transplants was defined as the “correct intervention rate” (CIR). The percent of recovered delisted patients that the model also decided to delist was summarized as the “correct removal rate” (CRR). For candidates who recovered, we assessed whether they were still delisted, potentially at an earlier timepoint. Given that such metrics reflect the identification of counterfactual actions, their absolute values were less important than the insight they provided in exposing deviation from real-life decisions.

In the decision of selecting an “optimal” model, we used the metrics that best embodied the goals of clinicians. The main objectives were to simultaneously maintain a low number of transplants, avoid the transplant decisions that led to graft failures, and maintain the transplants that led to (or could lead to) successful transplants. Concretely, we selected an optimal model that maintained a high GFPR, a high GSPR, and a low (but non-zero) number of transplant actions.

In addition to the clinically-focused metrics, we also utilized more conventional measures in RL to assess overfit during training. We observed the temporal-difference loss, conservative loss, and average value estimation over the duration of the training process^74,75^. Indicators of overfit included converging loss with diverging value estimations, suggesting that the model learned to overestimate out-of-distributions action-values. Ideally, the loss should decrease with a slow increase in value estimation. We observed this trend with $\alpha =0.1$, while also observing signs of overestimation with lower *α* and underfitting with higher *α –* all indicative of the utility of CQL and the existence of an optimal *α* that balances unseen value estimation and conservation.

While other popular metrics exist for off-policy evaluation, we elected for more clinically-driven metrics to improve interpretability and correspondence to clinical goals, which is desired in the field of RL for healthcare^62,76^. For example, weighted importance sampling (WIS) is used to provide an estimation of the value of a target with respect to the true behavior policy. However, in practice, the behavior policy is often unknown and dependent on additional estimations through methods such as regression or behavior cloning to learn a distribution of actions on the states^62,76,77^. For example, we demonstrate superiority of the CQL model with respect to different behavior policies when measuring performance with WIS (Supplementary Table 6). But even with an ideal behavior policy, interpretation is challenging when trajectories deviate significantly, and thus evaluation is difficult in complex problems such as donor-recipient matching^62,76,78^. Instead, inspired by WIS, we quantified the extent to which decisions matched the actions recorded in the data, but agnostic to the outcomes and value estimation of the counterfactual decisions; we defined the “action match rate” as the percent of total actions from the model that matched the decisions made in real-life. We also summarized the counts of actions across the holdout dataset to provide insight into the overall tendencies of each model.

### Baseline comparisons to a MELD threshold

We compared against naïve models denoted by “MELD *k*” where *k* represents a predefined MELD score threshold. These models simulated the scenario of an organ being transplanted to a candidate any time their MELD score exceeded the threshold. Given that there is currently no true reference for donor-recipient matching over time, we used the MELD score as it is the existing standard for candidate prioritization. While deviating from reality, the MELD threshold models provided an important reference to a decision-making process that only used candidate disposition and predictions of waitlist mortality without considering the quality of the donor-recipient pair or the longitudinal trajectory of the candidate. Multiple thresholds of $k=\left\{15,\,20,\,24,\,30\right\}$ were used to encapsulate a spectrum of conservative to non-conservative models based solely on MELD score. These thresholds were chosen primarily from a clinical expertise, as a MELD of 15 is the minimum score typically needed for placement on the LT waitlist and above 30 is considered high-risk for 90-day waitlist mortality. A MELD of 24 was the median score in the study population, while a score of 20 was chosen arbitrarily as an additional point of granularity to observe model behavior when forced to transplant between the high and low thresholds.

### Baseline comparisons to static predictive models

We also contrasted the RL approach against more traditional static predictive models that focused on the supervised learning of output variables. These models employed pattern recognition to map input features to output values at single timepoints, without considering the short- and long-term risks that are characteristic of the RL approach. We used multivariate logistic regression (LGR) as a simpler statistical method and extreme gradient boosting (XGB) as a more complex nonlinear model^79^. For each architecture, we trained three separate models with different objectives. First, we used actions as the dependent variable to learn “action prediction models” that predict actions given states. Next, we trained on terminal outcomes (i.e., successful transplant, delist recovery, graft failure/mortality), representing an “outcome prediction model” that predicts outcomes supervised using only the true, observable data. Lastly, we limited the dataset to the donor-recipient matches that occurred in real life, along with their corresponding outcomes, to create “graft failure prediction models” that illustrate the limitations of approaches in the literature that only focus on predicting graft failure. All predictive models used the same dataset splits and hyperparameter tuning pipeline as the RL approach.

### Feature comparisons

To statistically compare features, we used the two-sided Student’s *t*-test/Mann–Whitney U test for normally/non-normally distributed continuous variables (using the Shapiro–Wilk test to test for normality), the Fischer exact test for discrete binary variables, and the Chi-squared test for discrete multi-category variables. Effect sizes were respectively calculated using Hedge’s g, Cohen’s h, and Cramer’s V.

### Evaluation in a constrained setting

In the primary analysis, it was possible for the same donor to be offered to multiple candidates even if a decision was made to transplant it. Decisions made on a single candidate in their trajectory would also be independent of all other decisions, meaning that a candidate could be chosen to be delisted or transplanted at one point and subsequently could be transplanted or delisted again. This paradigm was necessary during training to preserve independence assumptions and during evaluation to force the model to make a decision on the terminal state that would include the true pair if the patient was transplanted. The analysis of the true pair was essential to assess the model’s decision-making on the real-life pairs that had associated outcomes.

As this approach deviates from reality, we designed a subsequent evaluation in a constrained setting that better reflects the decision-making process that occurs in real-life. We imposed a limitation that a donor could only be transplanted once and was removed as soon as they were transplanted. If a donor was matched with multiple candidates, then it was offered to the closest candidate in time. We also enforced that candidates were removed from the waitlist if they were transplanted or delisted and not eligible for any future decisions. The collected metrics included transplant and removal rates for the subgroups of candidates with different outcomes who were transplanted or delisted.

## Supplementary information

Supplement

## Data Availability

The data that support the findings of this study are available from the SRTR, which leverages an Internet-based system called UNet (SM) for data collection. Restrictions apply to the availability of these data, which were used under licence for the current study, and so are not publicly available. Data are however available upon reasonable request to the SRTR and compliance with their data use agreements. The corresponding code used in this work and examples are publicly available at https://github.com/uclamii/offline_liver_transplant.

## Code availability

The corresponding code used in this work and examples are publicly available at https://github.com/uclamii/offline_liver_transplant. To the best of our ability, we adhered to the TRIPOD-AI guidelines for the development of prediction models that use ML methods (Supplementary Table 7).
