# Supplementary material for: Liver transplant donor-recipient matching with offline reinforcement learning
Source: NPJ Digit Med. 2026 Mar 16;9:351. doi: 10.1038/s41746-026-02529-1 (PMC13136313; doi:10.1038/s41746-026-02529-1)
Supplement: Supplementary file 1 — Supplement [file 41746_2026_2529_MOESM1_ESM.pdf]

Liver Transplant Donor-Recipient Matching with Offline Reinforcement Learning

Andrew Melehy[1*†], Jeffrey Feng[1†], Dominic Amara[1], Vatche G. Agopian[1], Alex A. T. Bui[1]

[1]University of California Los Angeles, Los Angeles, United States

[*]Corresponding author(s). E-mail(s): amelehy@mednet.ucla.edu; Contributing authors: j64feng@g.ucla.edu; damara@mednet.ucla.edu; vagopian@mednet.ucla.edu; buia@mii.ucla.edu;
[†]These authors contributed equally to this work.

Supplementary Table 1. Descriptive demographics of LT candidates meeting study inclusion criteria

**Variable**	**Total cohort (n=45,635)**
Age, y (median [IQR])	56.00 [48.00, 63.00]
Sex = M (%)	27,494 (60.2)
Height, cm (median [IQR])	171.50 [162.60, 179.07]
Weight, kg (median [IQR])	83.70 [70.60, 98.88]
BMI (median [IQR])	28.00 [25.00, 33.00]
Liver Disease Etiology (%)	
Alcoholic Cirrhosis	19,044 (41.7)
Autoimmune	1,508 (3.3)
Biliary Disease	3,792 (8.3)
Cirrhosis – Other	527 (1.2)
Hepatitis B	597 (1.3)
Hepatitis C	2,978 (6.5)
Malignancy	1,144 (2.5)
Metabolic Disease	758 (1.7)
MASLD	10,708 (23.5)
Other	3,599 (7.9)
Diabetes (%)	12645 (27.7)
Blood Type, ABO (%)	
A	17,339 (38.0)
AB	1,813 (4.0)
B	5,402 (11.8)
O	21,081 (46.2)
Mechanical Ventilation (%)	808 (1.8)
Bacterial Peritonitis (%)	5,350 (11.7)
Portal Vein Thrombosis (%)	3,813 (8.4)
TIPSS (%)	4,040 (8.9)
Ascites (%)	
Absent	7,124 (15.6)
Moderate	15,804 (34.7)
Slight	22,676 (49.7)
Encephalopathy (%)	
Grade 1-2	27,127 (59.5)
Grade 3-4	4,658 (10.2)
None	13,820 (30.3)
Dialysis (%)	5,403 (11.8)
MELD listing (median [IQR])	19.00 [14.00, 27.00]
MELD last (median [IQR])	22.00 [15.00, 31.00]
INR listing (median [IQR])	1.57 [1.30, 2.08]
Sodium listing (median [IQR])	136.00 [132.00, 139.00]
Creatinine listing (median [IQR])	1.08 [0.80, 1.67]
Bilirubin listing (median [IQR])	3.50 [1.78, 8.60]
Albumin listing (median [IQR])	3.10 [2.70, 3.60]
Outcomes	
Transplanted (%)	28,587 (62.6)
Death or Delisting (%)	7,616 (16.7)
Delisting, recovered (%)	2,678 (6.2)

Supplementary Table 2. Demographic comparisons between deceased donors used in liver transplants and discarded donor organs

Variable	Used (n=26,790)	Discard (n=16,272)	P-value
Age, y (median [IQR])	40.00 [29.00, 53.00]	49.00 [37.00, 57.00]	<0.001
Blood Type, ABO (%)			<0.001
A	10186 (38.0)	6100 (37.5)	
AB	764 (2.9)	728 (4.5)	
B	3018 (11.3)	1889 (11.6)	
O	12822 (47.9)	7555 (46.4)	
Height, cm (median [IQR])	172.72 [165.00, 180.00]	172.00 [165.00, 178.00]	<0.001
Weight, kg (median [IQR])	80.90 [68.80, 95.10]	86.70 [72.60, 105.00]	<0.001
Creatinine (median [IQR])	1.10 [0.77, 1.84]	1.00 [0.70, 1.77]	<0.001
Blood urea nitrogen (median [IQR])	22.00 [15.00, 36.00]	22.00 [15.00, 35.00]	0.025
Total bilirubin (median [IQR])	0.60 [0.40, 1.00]	0.70 [0.40, 1.30]	<0.001
SGOT/AST (median [IQR])	41.00 [24.00, 82.00]	59.00 [33.00, 124.00]	<0.001
SGPT/ALT (median [IQR])	38.00 [21.00, 78.00]	46.00 [25.00, 101.00]	<0.001
Sodium (median [IQR])	148.00 [143.00, 154.00]	146.00 [141.00, 152.00]	<0.001
INR (median [IQR])	1.22 [1.10, 1.40]	1.20 [1.10, 1.40]	0.846
PH (median [IQR])	7.41 [7.37, 7.45]	7.40 [7.35, 7.45]	<0.001
Hematocrit (median [IQR])	28.10 [25.00, 32.00]	29.70 [25.60, 34.60]	<0.001
Cause of death (%)			<0.001
Anoxia	11920 (44.5)	7669 (47.1)	
Stroke	6939 (25.9)	4430 (27.2)	
Head Trauma	7289 (27.2)	3321 (20.4)	
CNS Tumor	86 (0.3)	45 (0.3)	
Other	556 (2.1)	807 (5.0)	
Anit-HIV I/II (%)	38 (0.1)	130 (0.8)	<0.001
Anti-HTLV I/II (%)	14 (0.1)	6 (0.0)	<0.001
RPR-VDRL (%)	324 (1.2)	194 (1.2)	<0.001
Anti-CMV (%)	16968 (63.3)	10070 (61.9)	0.001
HBsAg (%)	42 (0.2)	63 (0.4)	<0.001
Anti-HBC (%)	1352 (5.0)	1166 (7.2)	<0.001
Anti-HCV (%)	2591 (9.7)	2059 (12.7)	<0.001
HBsAb (%)	1204 (4.5)	686 (4.2)	<0.001
EBV IgG (%)	24103 (90.0)	14903 (91.6)	<0.001
EBV IgM (%)	364 (1.4)	276 (1.7)	<0.001
Clinical infection (%)	20646 (77.1)	11456 (70.4)	<0.001
Inotropic support (%)	10881 (40.6)	4481 (27.5)	<0.001
Infection source: blood (%)	3351 (12.5)	2459 (15.1)	<0.001
Cocaine use (%)	6383 (23.8)	3908 (24.0)	<0.001
% Micro vesicular fat (median [IQR])	5.00 [0.00, 10.00]	10.00 [0.00, 21.00]	<0.001
% Macro vesicular fat (median [IQR])	5.00 [0.00, 10.00]	20.00 [5.00, 45.00]	<0.001
Final MAP, mmHg (median [IQR])	88.00 [79.00, 98.00]	87.00 [78.00, 99.00]	0.530
Initial MAP, mmHg (median [IQR])	84.00 [73.00, 97.00]	81.50 [73.00, 92.00]	0.177
Maximum creatinine (median [IQR])	1.10 [0.78, 1.85]	1.01 [0.70, 1.77]	<0.001
High risk by CDC guideline (%)	7116 (26.6)	3805 (23.4)	<0.001
Donation after circulatory death (%)	2586 (9.7)	8713 (53.5)	<0.001

Supplementary Table 3. Definitions of metrics.

Name	Abbreviation	Description
Graft failure prevention rate	GFPR	Percent of candidates that experienced graft failure that the model decided not to transplant given their true pair
Graft failure intervention rate	GFIR	Percent of candidates that experienced graft failure the model decided to transplant to an earlier pair
Graft success preservation rate	GSPR	Percent of candidates that experienced successful transplant that the model decided to transplant given their true pair or an earlier pair
Correct removal rate	CRR	Percent of candidates that recovered on the waitlist that the model decided to delist at any time
Correct intervention rate	CIR	Percent of candidates that experienced waitlist mortality that the model decided to transplant before death
Action match rate	--	Percent of actions decided on by the model that matched the real-life actions recorded in the data

Supplementary Table 4. Stratified evaluation results for the final conservative Q-learning based offline reinforcement model stratified by demographic factors including: candidate sex, the location of the candidate defined by the 11 United Network for Organ Sharing (UNOS) geographic regions, and candidate race. Metrics are consistent with those reported in **Table 1**.

Variable	n (%)	Action match rate, % (SD)	Action: Wait, n (SD)	Action: Delist, n (SD)	Action: Transplant, n (SD)	GFPR, % (SD)	GFIR, % (SD)	GSPR, % (SD)	CRR, % (SD)	CIR, % (SD)
**Sex**										
Female	3376 (38.72)	73.86 (1.31)	14,186.80 (347.03)	423.20 (43.78)	2,681.00 (320.58)	73.46 (4.25)	55.47 (6.33)	92.84 (1.34)	38.48 (2.75)	47.98 (2.90)
Male	5343 (61.28)	72.51 (0.80)	20,660.60 (395.96)	617.00 (75.06)	4,246.40 (356.37)	73.00 (0.85)	54.30 (3.34)	93.24 (1.10)	36.92 (3.89)	45.93 (3.41)
**Region**										
1	450 (5.16)	74.87 (1.21)	2,201.80 (53.68)	107.80 (17.80)	393.40 (45.24)	74.44 (9.30)	55.56 (10.39)	95.05 (1.17)	45.84 (5.29)	52.04 (3.67)
2	1030 (11.81)	72.40 (0.80)	4,018.60 (92.86)	167.80 (16.33)	800.60 (78.46)	71.74 (5.95)	58.70 (5.10)	93.16 (1.66)	44.20 (3.59)	45.99 (2.24)
3	1291 (14.81)	71.91 (1.03)	4,610.40 (94.43)	106.60 (12.97)	941.00 (90.09)	74.32 (2.70)	53.51 (4.23)	92.89 (1.06)	33.39 (4.62)	45.71 (4.93)
4	1091 (12.51)	72.77 (0.90)	4,173.40 (70.82)	128.60 (10.81)	844.00 (63.02)	72.31 (3.34)	54.36 (9.66)	94.12 (1.68)	37.76 (3.55)	43.27 (4.07)
5	1368 (15.69)	74.01 (1.01)	6,136.40 (143.84)	180.60 (26.61)	1,214.00 (143.71)	77.31 (3.86)	51.94 (5.91)	93.64 (1.04)	38.64 (5.89)	53.36 (2.26)
6	216 (2.48)	73.67 (1.53)	904.80 (20.07)	19.00 (3.39)	207.20 (18.20)	77.78 (17.57)	51.11 (12.67)	97.07 (1.36)	37.50 (5.10)	51.00 (8.02)
7	686 (7.87)	73.57 (1.09)	2,817.00 (70.10)	62.60 (18.37)	596.40 (60.13)	62.93 (8.52)	58.05 (8.86)	93.15 (1.15)	31.63 (6.80)	54.46 (5.64)
8	471 (5.40)	72.71 (0.55)	1,642.80 (38.11)	35.60 (3.91)	318.60 (37.59)	76.36 (7.47)	38.18 (7.61)	93.75 (2.32)	27.76 (6.56)	39.66 (7.00)
9	473 (5.42)	73.07 (1.41)	2,086.80 (59.18)	70.60 (5.77)	450.60 (58.35)	75.45 (9.96)	59.09 (4.55)	93.20 (0.72)	45.23 (2.79)	55.11 (5.29)
10	811 (9.30)	71.59 (1.33)	2,872.80 (55.54)	94.80 (11.12)	491.40 (50.16)	74.36 (4.05)	44.62 (4.66)	88.72 (2.94)	38.96 (4.31)	34.43 (5.09)
11	832 (9.54)	73.60 (1.40)	3,382.60 (69.43)	66.20 (9.52)	670.20 (66.32)	71.56 (5.31)	60.00 (8.89)	93.73 (1.16)	28.94 (3.64)	42.03 (3.28)
**Race**										
Asian	261 (2.99)	71.17 (1.92)	996.40 (18.02)	40.00 (5.52)	218.60 (22.43)	80.00 (7.82)	48.57 (5.98)	89.35 (2.09)	39.13 (4.07)	47.76 (5.88)
Black	527 (6.04)	71.81 (1.47)	1,953.60 (53.39)	72.40 (14.81)	417.00 (51.05)	67.86 (6.19)	55.00 (5.42)	92.33 (1.46)	43.81 (5.45)	48.45 (6.73)
Other	149 (1.71)	74.51 (1.96)	620.60 (16.46)	19.40 (5.37)	121.00 (13.66)	64.00 (18.17)	56.00 (18.17)	96.05 (2.03)	40.00 (6.12)	46.96 (8.36)
White	7782 (89.25)	73.16 (0.91)	31,276.80 (648.00)	908.40 (100.83)	6,170.80 (586.96)	73.57 (2.37)	54.38 (3.11)	93.20 (1.18)	37.00 (3.51)	46.71 (2.80)

Supplementary Table 5. Summary of reward design.

Component	Final Coefficient	Description
$(MELD_i - MELD_{i+1}) \times \frac{min(MELD_{i+1}, 24)}{24}$	--	Decrease in MELD is a positive reward. Increase in MELD is a negative reward. The magnitude of MELD change was scaled by a factor of how close the MELD was to the median MELD score in the study population, which was 24.
$\beta_1 \mathbf{1}_{\text{GF donor}}(\boldsymbol{donor})$	1.0	Positive reward for waiting on potential donors that led to eventual graft failure.
$\beta_2 \mathbf{1}_{\text{Discarded donor}}(\boldsymbol{donor})$	0.25	Positive reward for waiting on potential donors that were eventually discarded.
β_3	-1.0	Negative reward for delisting due to medical deterioration or being too sick to transplant.
β_4	1.0	Positive reward for delisting for medical condition improvement.
β_5	-4.0	Negative reward for transplants resulting in a 1-year graft failure.
β_6	1.0	Positive reward for transplants resulting in a successful transplant.

Supplementary Table 6. Weighted importance sampling (WIS) metric of the optimal conservative Q-learning model with respect to different behavior policies.

Behavior Policy	WIS (SD)
Logistic regression	3.06 (0.41)
XGBoost	2.12 (0.69)
Behavior Cloning	1.27 (0.31)

Supplementary Table 7. TRIPOD-AI Checklist.

Section/Topic	Item	Checklist Item	Reported
Title	1	Identify the study as developing or evaluating the performance of a multivariable prediction model, the target population, and the outcome to be predicted	1
Abstract	2	See TRIPOD+AI for Abstracts checklist	2
Background	3a	Explain the healthcare context (including whether diagnostic or prognostic) and rationale for developing or evaluating the prediction model, including references to existing models	3
	3b	Describe the target population and the intended purpose of the prediction model in the context of the care pathway, including its intended users (e.g., healthcare professionals, patients, public)	3
	3c	Describe any known health inequalities between sociodemographic groups	--
Objectives	4	Specify the study objectives, including whether the study describes the development or validation of a prediction model (or both)	5
Data	5a	Describe the sources of data separately for the development and evaluation datasets (e.g., randomized trial, cohort, routine care or registry data), the rationale for using these data, and representativeness of the data	5
	5b	Specify the dates of the collected participant data, including start and end of participant accrual; and, if applicable, end of follow-up	5
Participants	6a	Specify key elements of the study setting (e.g., primary care, secondary care, general population) including the number and location of centers	5
	6b	Describe the eligibility criteria for study participants	5
	6c	Give details of any treatments received, and how they were handled during model development or evaluation, if relevant	5
Data preparation	7	Describe any data pre-processing and quality checking, including whether this was similar across relevant sociodemographic groups	6
Outcome	8a	Clearly define the outcome that is being predicted and the time horizon, including how and when assessed, the rationale for choosing this outcome, and whether the method of outcome assessment is consistent across sociodemographic groups	6
	8b	If outcome assessment requires subjective interpretation, describe the qualifications and demographic characteristics of the outcome assessors	6
	8c	Report any actions to blind assessment of the outcome to be predicted	6
Predictors	9a	Describe the choice of initial predictors (e.g., literature, previous models, all available predictors) and any pre-selection of predictors before model building	6
	9b	Clearly define all predictors, including how and when they were measured (and any actions to blind assessment of predictors for the outcome and other predictors)	6
	9c	If predictor measurement requires subjective interpretation, describe the qualifications and demographic characteristics of the predictor assessors	6
Sample size	10	Explain how the study size was arrived at (separately for development and evaluation), and justify that the study size was sufficient to answer the research question. Include details of any sample size calculation	5
Missing data	11	Describe how missing data were handled. Provide reasons for omitting any data	5
Analytical methods	12a	Describe how the data were used (e.g., for development and evaluation of model performance) in the analysis, including whether the data were partitioned, considering any sample size requirements	6,20
	12b	Depending on the type of model, describe how predictors were handled in the analyses (functional form, rescaling, transformation, or any standardization)	6
	12c	Specify the type of model, rationale, all model-building steps, including any hyperparameter tuning, and method for internal validation	14-20
	12d	Describe if and how any heterogeneity in estimates of model parameter values and model performance was handled and quantified across clusters (e.g., hospitals, countries)	12
	12e	Specify all measures and plots used (and their rationale) to evaluate model performance (e.g., discrimination, calibration, clinical utility) and, if relevant, to compare multiple models	18
	12f	Describe any model updating (e.g., recalibration) arising from the model evaluation, either overall or for particular sociodemographic groups or settings	--
	12g	For model evaluation, describe how the model predictions were calculated (e.g., formula, code, object, application programming interface)	23
Class imbalance	13	If class imbalance methods were used, state why and how this was done, and any subsequent methods to recalibrate the model or the model predictions	--
Fairness	14	Describe any approaches that were used to address model fairness and their rationale	--
Model output	15	Specify the output of the prediction model (e.g., probabilities, classification). Provide details and rationale for any classification and how the thresholds were identified	6
Training vs evaluation	16	Identify any differences between the development and evaluation data in healthcare setting, eligibility criteria, outcome, and predictors	12
Ethical approval	17	Name the institutional research board or ethics committee that approved the study and describe the participant-informed consent or the ethics committee waiver of informed consent	5
Funding	18a	Give the source of funding and the role of the funders for the present study	30
Conflicts of interest	18b	Declare any conflicts of interest and financial disclosures for all authors	30
Protocol	18c	Indicate where the study protocol can be accessed or state that a protocol was not prepared	--
Registration	18d	Provide registration information for the study, including register name and registration number, or state that the study was not registered	--
Data sharing	18e	Provide details of the availability of the study data	24
Code sharing	18f	Provide details of the availability of the analytical code	24
Patient & Public Involvement	19	Provide details of any patient and public involvement during the design, conduct, reporting, interpretation, or dissemination of the study or state no involvement	30
Participants	20a	Describe the flow of participants through the study, including the number of participants with and without the outcome and, if applicable, a summary of the follow-up time. A diagram may be helpful	--
	20b	Report the characteristics overall and, where applicable, for each data source or setting, including the key dates, key predictors (including demographics), treatments received, sample size, number of outcome events, follow-up time, and amount of missing data. Report any differences across key demographic groups	--
	20c	For model evaluation, show a comparison with the development data of the distribution of important predictors (demographics, predictors, and outcome)	--
Model development	21	Specify the number of participants and outcome events in each analysis (e.g., for model development, hyperparameter tuning, model evaluation)	5
Model specification	22	Provide details of the full prediction model (e.g., formula, code, object, API) to allow predictions in new individuals and to enable third-party evaluation and implementation	24
Model performance	23a	Report model performance estimates with confidence intervals, including for any key subgroups (e.g., sociodemographic). Consider plots to aid presentation	8
	23b	If examined, report results of any heterogeneity in model performance across clusters	--
Model updating	24	Report the results from any model updating, including the updated model and subsequent performance	--
Interpretation	25	Give an overall interpretation of the main results, including issues of fairness in the context of the objectives and previous studies	10
Limitations	26	Discuss any limitations of the study (such as a non-representative sample, sample size, overfitting, missing data) and their effects on any biases, statistical uncertainty, and generalizability	12-13
Usability of the model	27a	Describe how poor quality or unavailable input data should be assessed and handled when implementing the prediction model	13
	27b	Specify whether users will be required to interact in the handling of the input data or use of the model, and what level of expertise is required of users	13
	27c	Discuss any next steps for future research, with a specific view to applicability and generalizability of the model	13

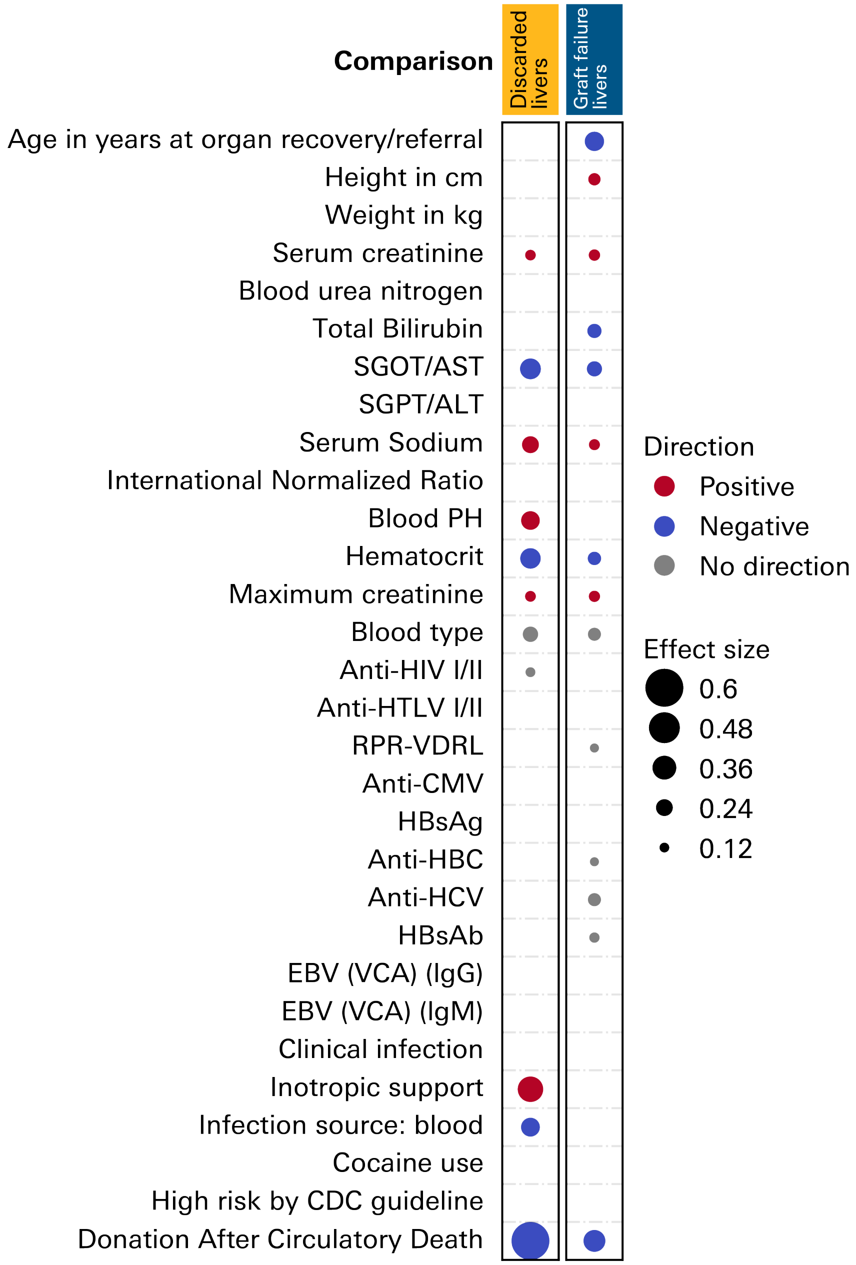

Supplementary Figure 1. Feature map comparing: discarded donor livers that were selected for transplant by the final CQL model to discarded livers that were not selected (left); and donor livers that led to true graft failures that were selected for transplant by the final CQL model to donor livers that led to true graft failures that were not selected (right).

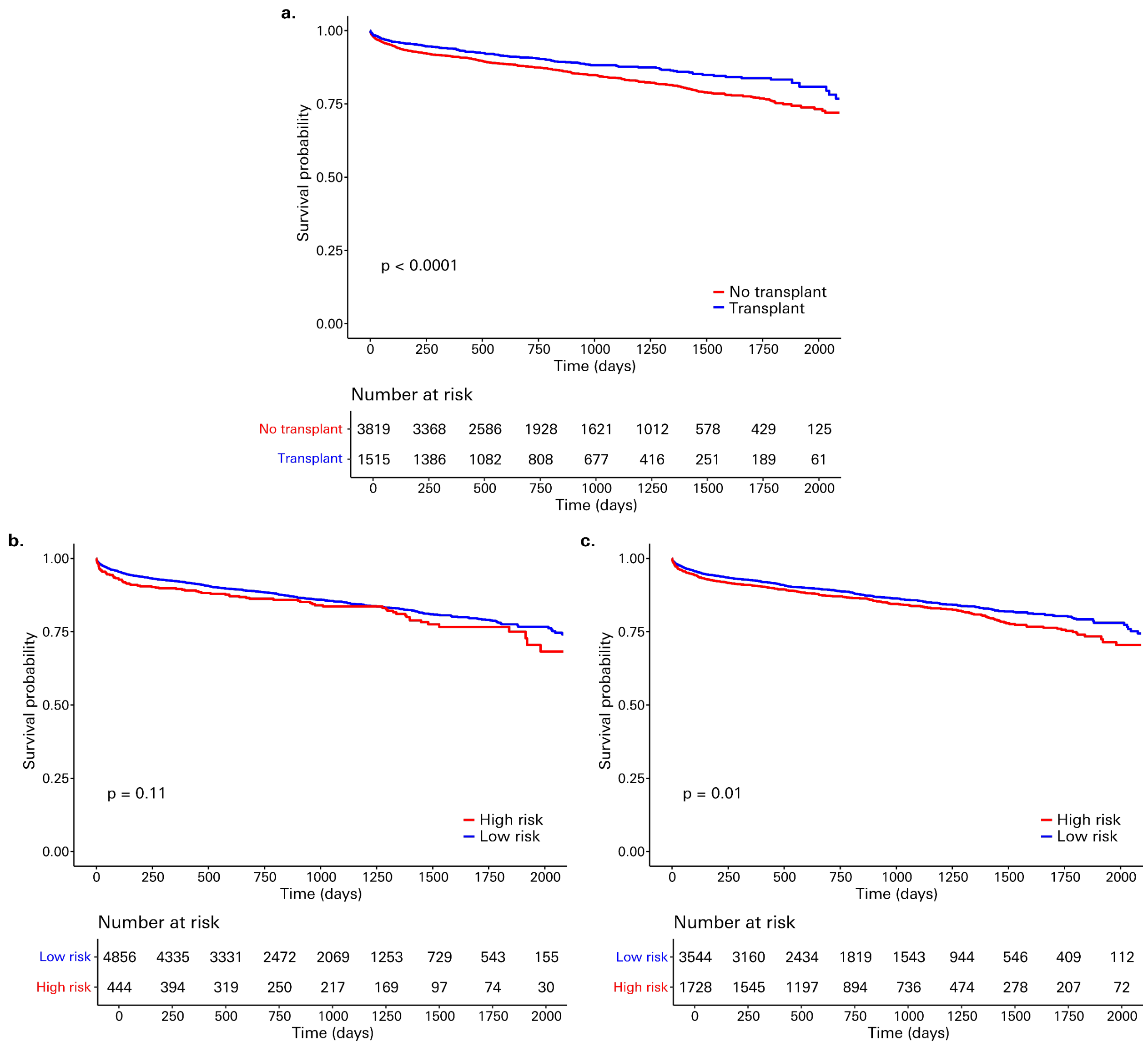

Supplementary Figure 2. Kaplan-Meier curves comparing post-transplant survival in: **a)** true donor-recipient pairs selected by the CQL model for transplant to those not selected; **b)** low and high-risk patients based on a BAR score threshold of 18; and **c)** low and high-risk patients based on SOFT score designations of high-moderate, high, and futile.

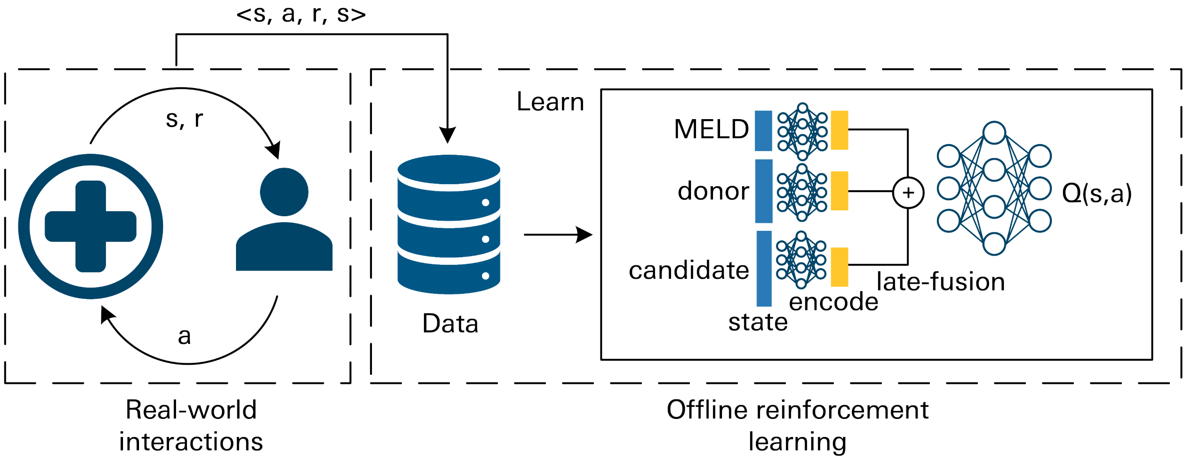

Supplementary Figure 3. Overview of the data collection and offline RL pipeline. In offline RL, data is collected from sampling real-world interactions between an agent and an environment. Trajectories are formed as the agents perform actions (*a*) in the environment, resulting in changes of state (*s*) and a reward (*r*). Transitions (*s, a, r, s*) are extracted from these trajectories to form the final dataset for offline RL. Here, we illustrate the general schematic of CQL – one approach to offline RL – that is based on deep Q-learning. An action-value function *Q* is parameterized by a deep neural network that learns the value given states and actions to ultimately choose the action that maximizes the expected return. The inputs to the *Q* function are the embedded observations pertaining to the potential donor-recipient pairs.

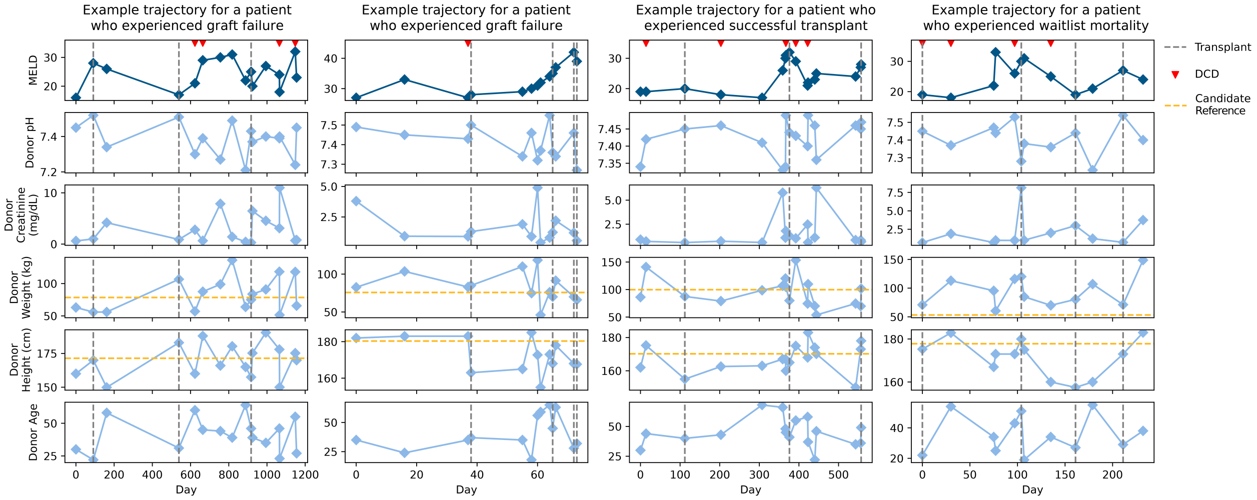

Supplementary Figure 4. Examples of patient trajectories throughout the donor-recipient matching process, and transplant decisions by the optimal CQL model. Transplant suggestions by the CQL model are indicated by the gray vertical lines. Note that these trajectories illustrate a simulated scenario where a candidate could be transplanted at multiple points in time, as a way to demonstrate the full waitlist period for a given patient. Realistically, they would be transplanted at their first indicated match. The two left columns illustrate patients who experienced graft failure. The second column to the right illustrates a patient who received a successful transplant. The rightmost column illustrates a patient who experienced waitlist mortality. Donations after circulatory death (DCD) donors are indicated by the red triangles. Select features are shown, including: candidate MELD, donor PH, donor creatinine, donor weight, donor height, and donor age over time.
